# Orthoform, “New,” as a Local Anesthetic, Analgesic and Antiseptic

**Published:** 1899-02

**Authors:** 


### Abstracts.

### ORTHOFORM, "NEW," AS A LOCAL ANESTHETIC, ANALGESIC AND ANTISEPTIC.

NORTH, professor of laryngology, Toledo Medical College, (*American Medical Compend*), says that he has used this compound in advanced pulmonary and laryngeal phthisis, in all forms and varieties of ulcerations of the nose, mouth, pharynx and larynx, after the removal of the faucial tonsils, and even in painful carious teeth, with the most gratifying results. He says after operating in the nasal cavities under the influence of cocaine, he applies orthoform freely, and as a result has no pain to contend with and the vessels do not dilate and hemorrhage does not return and the parts heal very rapidly.

After the removal of the faucial tonsils he applies orthoform to the cut surfaces, the patients can eat solid food without pain and the parts heal quickly. There is no pain after removal of an elongated uvula if orthoform is applied.

In all cases of painful laryngeal troubles in which he applies orthoform, the pain is relieved at once and the patient experiences a relief that lasts for hours or days.

He had a very painful carious tooth. Cocaine would relieve the pain for a short time. He packed the cavity with orthoform and had no pain in the tooth for a week.

After giving details of cases and conditions in which the preparation was used, he concludes as follows : This is about the extent of my use of orthoform. I am convinced that it is a very valuable remedy in removing pain when applied to nerve endings. Its antiseptic and healing properties are also of very great value. It is a remedy that I could not dispense with in my practice without very great inconvenience.

